# Visualizing the Choriocapillaris Under Drusen: Comparing 1050-nm Swept-Source Versus 840-nm Spectral-Domain Optical Coherence Tomography Angiography

**DOI:** 10.1167/iovs.15-18915

**Published:** 2016-08-17

**Authors:** Mark Lane, Eric M. Moult, Eduardo A. Novais, Ricardo N. Louzada, Emily D. Cole, ByungKun Lee, Lennart Husvogt, Pearse A. Keane, Alastair K. Denniston, André J. Witkin, Caroline R. Baumal, James G. Fujimoto, Jay S. Duker, Nadia K. Waheed

**Affiliations:** 1 New England Eye Center Tufts Medical Center, Boston, Massachusetts, United States; 2 Queen Elizabeth Hospital Birmingham, University Hospitals Birmingham NHS Foundation Trust, Birmingham, United Kingdom; 3 Department of Electrical Engineering and Computer Science, and Research Laboratory of Electronics, Massachusetts Institute of Technology, Cambridge, Massachusetts, United States; 4 Federal University of São Paulo, School of Medicine, São Paulo, Brazil; 5 Federal University of Goiás, Goiânia, Brazil; 6 Pattern Recognition Lab, Friedrich-Alexander University Erlangen-Nürnberg (FAU), Erlangen-Nürnberg, Germany; 7 NIHR Biomedical Research Center for Ophthalmology, Moorfields Eye Hospital NHS Foundation Trust and UCL Institute of Ophthalmology, London, United Kingdom; 8 Birmingham & Midland Eye Center, Sandwell and West Birmingham NHS Trust, Birmingham, United Kingdom

**Keywords:** optical coherence tomography, angiography, age-related macular degeneration, choriocapillaris

## Abstract

**Purpose:**

To investigate the appearance of choriocapillaris (CC) flow under drusen by comparing long-wavelength (1050 nm) swept-source optical coherence tomography (SS-OCT) angiography with shorter-wavelength (840 nm) spectral-domain (SD) OCT angiography.

**Methods:**

Patients with drusen imaged on both devices on the same day were selected and graded. Ambiguous OCT angiography (OCTA) signal loss was defined as low OCTA signal on the en face OCTA CC image that also had low OCT signal in the corresponding area on the en face OCT CC image and OCT B-scans. Unambiguous OCTA signal loss was defined as low OCTA signal on the en face OCTA CC image that did not have low OCT signal in the corresponding area on the en face OCT CC image and OCT B-scans. False-positive flow impairment on SS-OCTA was defined as ambiguous OCTA signal loss on SS-OCTA but no OCTA signal loss on SD-OCTA. False-positive flow impairment on SD-OCTA was defined as ambiguous OCTA signal loss on SD-OCTA but no OCTA signal loss on SS-OCTA.

**Results:**

Nine eyes from seven patients were enrolled, 23 drusen were analyzed. On 840-nm SD-OCTA, 17 drusen (73.9%) exhibited OCTA signal loss. Fourteen (82.4%) were classified as ambiguous, and three (17.6%) were classified as unambiguous; 10 (58.8%) were classified as having false-positive flow impairment. On 1050-nm SS-OCTA, seven drusen (30.4%) exhibited OCTA signal loss and were classified as unambiguous; none were classified as having false-positive flow impairment.

**Conclusions:**

Results showed that 1050-nm SS-OCTA appears less prone to producing areas of false-positive flow impairment under drusen.

Age-related macular degeneration (AMD) is the leading cause of irreversible blindness in adults older than 50 years in developed countries.^1–3^ Globally, AMD accounts for 8.7% of all blindness and is predicted to affect 196 million people by 2020.^4^ Early and intermediate AMD is characterized by soft drusen, which can be observed by fundoscopic examination as yellow deposits that lie between the retinal pigment epithelium (RPE) and the anterior collagenous layer of Bruch's membrane.^5,6^

The choroid is an extensive vascular network, consisting of an outer macrovascular layer and an inner capillary layer called the choriocapillaris (CC), which lies immediately below Bruch's membrane. The choroidal circulation provides 70% of the total blood flow to the eye, providing nutrients and removing waste products from the outer retinal structures.^7,8^ Histopathologic studies have implicated CC loss as a risk factor for the development of AMD.^8–13^ Many articles have indicated that the site of drusen formation is not random, but rather is influenced by CC dysfunction. In particular, it has been suggested that drusen are more likely to form at sites of insufficient choroidal perfusion, secondary to vascular endothelial cell loss.^14–17^ It is for this reason that drusen formation is often used as a surrogate marker for CC dysfunction.

Clinically, fluorescein angiography (FA) and indocyanine green angiography (ICGA) are considered the gold standards for imaging the retinal and choroidal vasculatures.^18–20^ Prolonged choroidal filling is a common feature of early AMD on both FA and ICGA, and indicates a vascular insufficiency that has a predilection for sites underlying drusen.^21^ Unfortunately, the microvascular architecture is often obscured by subsequent hyperfluorescence in the late phase of dye transit, which can limit precise assessment of CC loss and dysfunction in AMD. In addition, these modalities are invasive, involving the use of intravenous contrast that can result in systemic side effects, such as nausea, vomiting, and, rarely, anaphylaxis.^22–24^

Optical coherence tomography angiography (OCTA) is a noninvasive modality that generates depth-resolved images of the ocular vasculature by acquiring repeated B-scans from the same retinal location.^25–31^ The OCTA signal describes the pixel-by-pixel changes that occur in the repeated B-scans as a result of erythrocyte movement. Specifically, if the imaged tissue is stationary, the repeated B-scans will be identical, and no OCTA signal will be generated. Conversely, if the tissue contains flowing erythrocytes, the repeated B-scans will exhibit an intensity time variation that can be extracted and displayed.

Spectral-domain OCTA (SD-OCTA) provides excellent visualization of the retinal vasculature and also allows for visualization of the choroidal vasculature. However, the shorter, ∼840-nm, center wavelength typically used in commercial SD-OCT systems is strongly attenuated by the RPE; this attenuation can become more severe in the presence of RPE clumping and drusen. Furthermore, the spectrometer-based detection used in SD-OCT is susceptible to so-called sensitivity roll-off, the phenomenon whereby the sensitivity to retinal features farther from the system's zero-delay is lower than for retinal features that are closer to the system's zero-delay. When using the vitreoretinal imaging protocol with SD-OCT, sensitivity roll-off reduces the signal levels detected from the choroid region; CC imaging protocols with the SD-OCT system, whereby the CC is located near the top of the B-scan, closer to the system's zero-delay, do not suffer from sensitivity roll-off. Both RPE attenuation and sensitivity roll-off can result in low signal areas in choroidal regions, especially in areas below drusen.

Compared to shorter-wavelength SD-OCT, our prototype swept-source OCT (SS-OCT) system offers two distinct advantages: first, the longer, ∼1050-nm, center wavelength used in our SS-OCT systems is less attenuated by the RPE^32,33^; second, SS-OCT does not use spectrometer-based detection and is less susceptible to sensitivity roll-off. In particular, sensitivity roll-off in SS-OCT systems is determined by the instantaneous linewidth of the swept light source, along with the A/D acquisition. Together these two characteristics make SS-OCT less susceptible to low signal in regions below the RPE.

In the context of OCTA, areas of low OCT signal, which are heavily influenced by noise, produce unreliable, typically large, OCTA signals. Because of this, OCTA images are thresholded on the basis of the OCT signal level: OCTA pixels whose corresponding OCT signal level is below a predefined threshold are set to zero. This thresholding process prevents low OCT signal areas, such as the vitreous, from exhibiting a high OCTA signal, which is typically interpreted as corresponding to blood flow. However, thresholding OCTA images causes areas of low OCT signal to appear black on OCTA, irrespective of their blood flow status. Thus, the thresholding process introduces an ambiguity: it is no longer possible, from looking at the OCT and OCTA images alone, to ascertain if an area of low OCTA signal corresponds to low flow, low OCT signal, or both. Consequentially, thresholded OCTA images must be interpreted with care, and OCTA should always be viewed with the corresponding OCT so that OCT signal levels can be assessed.

The purpose of this study was to test the hypothesis that longer-wavelength, 1050-nm, SS-OCTA is less prone to producing areas of false-positive flow impairment than shorter-wavelength, 840-nm, SD-OCTA when imaging the CC underneath drusen.

## Methods

This was a retrospective case series conducted at the New England Eye Center (NEEC) of Tufts Medical Center, Boston, Massachusetts, and approved by the Tufts Medical Center and Massachusetts Institute of Technology Institutional Review Boards. The research adhered to the tenets of the Declaration of Helsinki and complied with the Health Insurance Portability and Accountability Act of 1996. Written informed consent was obtained before OCTA imaging.

### Patient Selection

Patients with drusen secondary to nonexudative AMD were seen at the retina service of NEEC between August 2014 and November 2015 and were recruited to be imaged on SD-OCT and SS-OCT. The diagnosis of AMD was confirmed by a retinal specialist on the basis of a complete ophthalmic evaluation including dilated fundus examination, standard structural SD-OCT imaging, and FA and/or ICGA as clinically indicated. Patients with drusen that could be clearly visualized and delineated on en face OCT and corresponding B-scan images were included. Large, soft drusen, ≥165 μm, were selected for analysis by a staff member from the Boston Image Reading Center who did not participate in image grading. Two trained readers from the Boston Image Reading Center reviewed and graded all OCT images. One eye was excluded from the analysis, as both readers agreed that the image quality was too poor to allow accurate grading.

### Image Acquisition and Analysis

All patients were imaged on SD-OCT and SS-OCT on the same day. The SD-OCT instrument was the RTVue XR Avanti (Optovue, Inc., Fremont, CA, USA) with prototype AngioVue software (software version 2015.0.0.69) for OCTA. This instrument operates at ∼840-nm wavelength and performs 70,000 A-scans per second to acquire OCTA volumes consisting of two repeated B-scans from 304 uniformly spaced locations. Each B-scan consists of 304 A-scans for a total of 2 × 304 × 304 A-scans per acquisition, with a total acquisition time of approximately 3 seconds, and an axial optical resolution of ∼5 μm. The interscan time is ∼5 ms. Split-spectrum amplitude-decorrelation angiography (SSADA) is used to improve the signal-to-noise ratio.^34^ Motion correction is performed by using coregistration of two orthogonally acquired volumes.^35,36^

The SS-OCT device was an ultrahigh speed, 400-kHz A-scan rate, prototype developed at Massachusetts Institute of Technology (Cambridge, MA, USA) and deployed to NEEC. It uses a high-speed vertical cavity surface emitting laser as the light source and operates at ∼1050 nm. A total of five repeated B-scans from 500 sequential, uniformly spaced locations are acquired. Each B-scan consists of 500 A-scans, and the interscan time is ∼1.5 ms. A total of 5 × 500 × 500 A-scans are acquired per OCTA volume with an acquisition time of ∼3.8 seconds, and an axial optical resolution of ∼8 to 9 μm.^37^ Motion correction, using the same algorithm as on the SD-OCT system, was performed. OCTA images were generated by using an intensity-based pixelwise decorrelation scheme of the form presented in the study by Jia et al.^34^; however, the spectrum was not split. To reduce noise in the SS-OCTA images, thresholding was performed. The threshold levels were computed, using the SS-OCT images, by estimating the mean and standard deviation of the noise, and then setting the threshold as 2 standard deviations above the noise mean.

For both systems, the 3 × 3 mm acquired OCT volumes were centered on the fovea. Furthermore, for both systems, images were obtained by using the vitreoretinal imaging protocol, which is the default setting in our clinic. The patients were asked to fixate on an internal target during OCTA acquisition. Drusen were identified by using OCT B-scans and the patients' fundus images. These were then correlated with the 3 × 3 mm OCTA images from both devices by two independent and masked readers of the Boston Image Reading Center.

The SD-OCT data were flattened by using the built-in automatic CC segmentation. The segmentation slab was manually adjusted by using corresponding structural OCT B-scans to align the segmentation with Bruch's membrane. For the SS-OCT data, a custom C++ application was used for processing, and ImageJ (http://imagej.nih.gov/ij/; provided in the public domain by the National Institutes of Health, Bethesda, MD, USA) was used for visualization. The SS-OCT data were flattened by using an automatic CC segmentation. As with the SD-OCT data, the segmentation slab was manually adjusted by using corresponding structural OCT B-scans to align the segmentation with Bruch's membrane. For both systems, en face OCT and OCTA CC slabs were formed by mean projecting the OCT and OCTA data, respectively, from 30 to 60 μm below the segmented line. Note that for the version of the Avanti software (2015.0.0.69) available at the time of this study, mean projection was the default setting.

**Figure 1:**
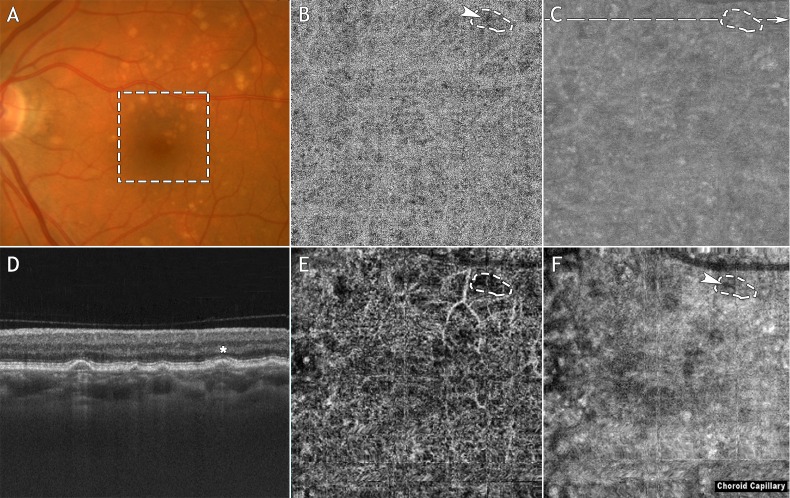
Example of OCTA signal loss on both the SD-OCT and SS-OCT systems. Note that this figure is intended to illustrate the differences in the SD-OCTA and SS-OCTA images and is not intended to describe the grading process itself. (**A**) Fundus photograph. (**B**) En face SS-OCTA CC slab. (**C**) En face SS-OCT CC slab. (**D**) Swept-source OCT B-scan extracted from the *dashed line* in (**C**). (**E**) En face SD-OCTA CC slab. (**F**) En face SD-OCT CC slab. The *dashed box* in (**A**) indicates the 3 × 3 mm field of view over which the SS-OCT and SD-OCT scans were acquired. In (**D**), a representative druse is denoted with an *asterisk*. The *dashed contour* in (**E**) highlights an area of OCTA signal loss underlying this drusen. The same contour has been superposed on images **B**, **C**, and **F**. Note that there is an area of SS-OCTA signal loss, indicated by the *arrowhead* in (**B**). Since the corresponding region in (**C**) does not exhibit low OCT signal, we classified the SS-OCTA signal loss in (**B**) as unambiguous. Since the *dashed contour* in (**F**) encircles a region of low SD-OCT signal, we classified the SD-OCTA signal loss in (**E**) as being ambiguous. Finally, neither the SD-OCTA nor the SS-OCTA is classified as having false-positive flow impairment.

Ambiguous OCTA signal loss was defined as an area of low OCTA signal on the en face OCTA CC slab that also had low OCT signal in the corresponding area on the en face OCT CC slab and associated OCT B-scans (Figs. 1E, 1F); ambiguous OCTA signal loss may be due to flow alteration, low OCT signal (e.g., caused by RPE attenuation), or both. We refer to these areas as ambiguous because it is not possible, in the absence of additional information, to determine whether the OCTA signal loss is caused by true CC alteration or is an artifact of the thresholding process. Unambiguous OCTA signal loss was defined as an area of low OCTA signal on the en face OCTA CC slab that did not have low OCT signal in the corresponding area on the en face OCT CC slab and associated OCT B-scans (Figs. 1B, 1C). We refer to these areas as unambiguous OCTA signal loss because the OCTA signal corresponding to such areas is unaffected by the thresholding function, that is, it is reflective of blood flow. This technique for assessing the cause of low OCTA signal in the CC has previously been described.^37^ False-positive flow impairment on SS-OCTA was defined as ambiguous OCTA signal loss on SS-OCTA but no OCTA signal loss on SD-OCTA; this suggests an SS-OCTA masking artifact, not true CC alteration. False-positive flow impairment on SD-OCTA was defined as ambiguous OCTA signal loss on SD-OCTA but no OCTA signal loss on SS-OCTA; this suggests an SD-OCTA masking artifact, not true CC alteration.

### Grader Analysis

Two observers independently analyzed the en face OCTA CC slabs from the SS-OCT and SD-OCT instruments. The presence or absence of low OCTA signal underlying the drusen was recorded. In patients where there was low OCTA signal underneath drusen, the images were correlated with the en face OCT CC slabs and OCT B-scans to classify the region as either corresponding to ambiguous OCTA signal loss or unambiguous OCTA signal loss.

Images were graded by two masked independent readers of the Boston Image Reading Center (ML and EAN). Interreader reliability was 97.8% (*κ* = 0.957, 95% CI: 0.872–1.00). The one image in which there was disagreement about low OCTA signal was arbitrated by another retinal specialist (RNL) to provide the final decision.

## Results

### Patient Demographics

Nine eyes from seven Caucasian patients with drusen secondary to AMD were enrolled in this retrospective study. Patients were classified as having dry AMD after an ophthalmologic examination and OCT. Patients with advanced AMD that had undergone FA examination with choroidal neovascularization were excluded from this study. Five patients (71.4%) were female and two (28.6%) were male. The mean age of the studied population was 73.1 ± 8.0 years (range, 57.5–87.3 years).

### Qualitative Analysis

En face OCTA CC slabs underneath 23 drusen were analyzed using both devices.

#### Spectral-Domain OCTA Analysis.

**Figure 2:**
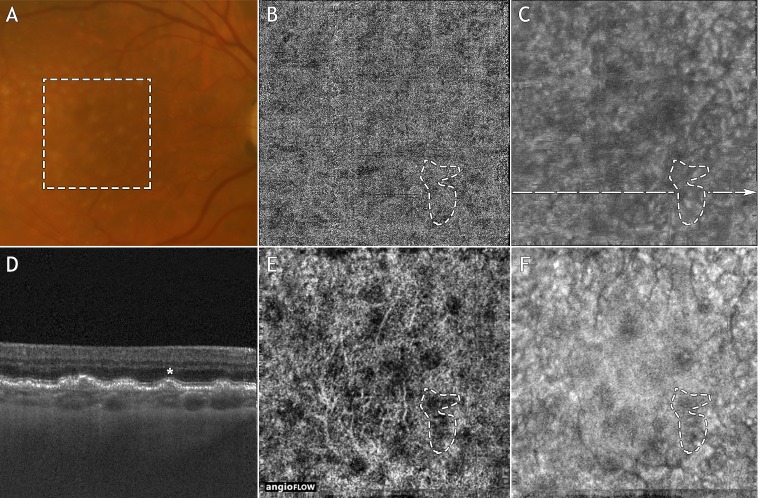
Example of OCTA signal loss on the SD-OCT system, and no OCTA signal loss on the SS-OCT system. Note that this figure is intended to illustrate the differences in the SD-OCTA and SS-OCTA images, and is not intended to describe the grading process itself. (**A**) Fundus photograph. (**B**) En face SS-OCTA CC slab. (**C**) En face SS-OCT CC slab. (**D**) Swept-source OCT B-scan extracted from the *dashed line* in (**C**). (**E**) En face SD-OCTA CC slab. (**F**) En face SD-OCT CC slab. The *dashed box* in (**A**) indicates the 3 × 3 mm field of view over which the SS-OCT and SD-OCT scans were acquired. In (**D**), a representative druse is denoted with an *asterisk*. The *dashed contour* in (**E**) highlights an area of OCTA signal loss underlying this drusen. The same contour has been superposed on images **B**, **C**, and **F**. Note that there is no SS-OCTA signal loss within the *dashed contour* of (**B**). Since the *dashed contour* in (**F**) encircles a region of low SD-OCT signal, we classified the SD-OCTA signal loss in (**E**) as being ambiguous. Finally, since there is no SS-OCTA loss in (**B**), we classified the SD-OCTA image as having false-positive flow impairment under this druse.

On the SD-OCTA device, 17 of the drusen (73.9%) exhibited underlying OCTA signal loss on the en face SD-OCTA CC slabs. Fourteen of these areas of OCTA signal loss (82.4%) were classified as ambiguous based of the low OCT signal levels in the corresponding en face SD-OCT CC slabs and SD-OCT B-scans (Figs. 2E, 2F). Three of these areas of OCTA signal loss (17.6%) were classified as unambiguous. Ten of the 17 areas of OCTA signal loss (58.8%) were classified as having false-positive flow impairment.

#### Swept-Source OCTA Analysis.

On the SS-OCTA device, 7 of the 23 drusen (30.4%) exhibited underlying loss of the OCTA signal. All seven of these areas of OCTA signal loss were classified as unambiguous; correspondingly, no areas were classified as having false-positive flow impairment (Figs. 2B, 2C).

## Discussion

Optical coherence tomography angiography is a noninvasive technique for rapidly acquiring depth-resolved images of the retinal and choroidal vasculature. Early and intermediate AMD is often characterized by the presence of drusen, and histopathologic studies have indicated that drusen may form in areas of CC insufficiency.^14–17^

Our current understanding of AMD has been hindered by the absence of a modality capable of adequately visualizing the CC. Notably, both of the gold-standard imaging modalities, FA and ICGA, have inherent flaws in their ability to image the CC. The image quality of FA is reduced by the absorption of the blue–green excitation wavelength of fluorescein by macular xanthophyll and the RPE. The fine microvascular network of the choroid is further obscured by leakage of ∼20% of the fluorescein dye that fails to bind to albumin, causing an early hyperfluorescence.^38^ Conversely, ICGA, which is considered the superior modality for imaging the choroid, has not gained widespread acceptance, as it is not depth resolved, so separating the CC blood flow from the deeper choroidal vasculature is a complex task.^39,40^

Because it does not suffer from the same limitations of dye-based techniques, OCTA is poised to establish itself as the standard modality for CC imaging. However, despite its advantages, OCTA images must be interpreted with care owing to a variety of image artifacts. As previously mentioned, OCTA images are typically thresholded on the basis of the OCT signal level: OCTA pixels whose corresponding OCT signal level is below a predefined threshold are set to zero. This thresholding step means that an area having both low OCTA signal and low OCT signal is ambiguous: the low OCTA signal may be the result of low flow, low OCT signal, or both. As OCT signal attenuation is likely to occur at depths below the RPE and underneath drusen, especially for shorter-wavelength systems, OCTA of the CC underneath drusen is particularly susceptible to such attenuation artifacts.^41^ To the authors' knowledge, a direct comparison of SS-OCTA and SD-OCTA for visualizing the CC underneath drusen has not been conducted to date.

Our study indicated that there were fewer drusen with areas of OCTA signal loss on the longer-wavelength, 1050-nm SS-OCTA system (*n* = 7) than on the shorter-wavelength, 840-nm SD-OCTA system (*n* = 17). Furthermore, 10 of the 17 areas of OCTA signal loss on SD-OCTA were classified as having false-positive flow impairment. In comparison, no areas on SS-OCTA were classified as having false-positive flow impairment. These results suggest that, when imaging the CC underlying drusen, longer-wavelength SS-OCTA is less prone to producing thresholding artifacts than shorter wavelength SD-OCTA. Regardless of the system used, in areas of low OCT signal, loss of OCTA signal should not be interpreted as CC flow alteration.

When interpreting the results of our study it is important to note that there are several differences between the two systems beyond the fact that one is a spectral-domain system and the other is a swept-source system. First, the wavelengths used are different: the SD-OCT system uses a shorter, 840-nm, light source, while the SS-OCT system uses a longer, 1050-nm, light source. Second, the underlying algorithms used to generate the OCTA images are different: the SD-OCT system uses the SSADA algorithm, while the SS-OCT system uses a different, custom, decorrelation-based approach. In addition, in any OCTA system, there are numerous processing and display options, ranging from thresholding levels, to contrast and brightness settings. Since the SD-OCT was a commercial system with proprietary software, it was not possible to equalize all of these settings between the two systems. Thus, there is the potential that some portion of our observations was due to such differences. However, we suspect that the primary factors underlying the observed results are a combination of signal attenuation, due to the wavelength difference, and sensitivity roll-off, due to the spectral-domain detection. With reference to the latter, it is also important to consider that imaging in our study was performed by using the vitreoretinal protocol, which is the default setting in our clinic. If the imaging had been performed in a configuration optimized for CC imaging, where the CC is near the top of the B-scan, closer to the zero-delay, the signal loss due to sensitivity roll-off would have been prevented. However, we opted to adhere to the vitreoretinal protocol because that is the standard imaging configuration that is used in our clinics; that SD-OCT requires different imaging protocols for the CC and retinal vasculatures in order to mitigate sensitivity roll-off is, in itself, a limitation of the SD-OCT.

There were several other important limitations to our study. Our sample size was small and did not cover a range of drusen types; we only selected large, soft drusen, ≥165 μm. It is possible that our results do not extend to different drusen types. Another limitation was that our analysis of OCT and OCTA signal levels was qualitative, not quantitative. While quantitative analysis would have been the most definitive route, because of the many unknown parameters of the commercial system, it was not feasible. In particular, processing parameters, such as thresholding levels, must be known to perform a fair quantitative comparison.

## Conclusions

This study suggests that, when imaging the CC beneath drusen, longer-wavelength, 1050-nm, SS-OCTA is less prone to producing areas of false-positive flow impairment than shorter-wavelength, 840-nm, SD-OCTA. Perhaps more importantly, this study underscores the need for careful interpretation of OCTA data, as there may be multiple factors that cause OCTA signal loss.
